# 5-Acetyl-4-(4-methoxy­phen­yl)-6-methyl-3,4-dihydro­pyrimidine-2(1*H*)-thione

**DOI:** 10.1107/S160053680904639X

**Published:** 2009-11-07

**Authors:** N. Anuradha, A. Thiruvalluvar, K. Pandiarajan, S. Chitra, R. J. Butcher

**Affiliations:** a PG Research Department of Physics, Rajah Serfoji Government College (Autonomous), Thanjavur 613 005, Tamil Nadu, India; b Department of Chemistry, Annamalai University, Annamalai Nagar 608 002, Tamil Nadu, India; c Department of Chemistry, Howard University, 525 College Street NW, Washington, DC 20059, USA

## Abstract

In the title mol­ecule, C_14_H_16_N_2_O_2_S, the heterocyclic ring adopts an envelope conformation with the plane through the five coplanar atoms making a dihedral angle of 88.99 (4)° with the benzene ring, which adopts an axial orientation. The thionyl, acetyl and methyl groups all have equatorial orientations. Inter­molecular N—H⋯S, N—H⋯O, C—H⋯O and C—H⋯S hydrogen bonds are found in the crystal structure.

## Related literature

For related crystal structures and their chemical and biological applications, see: Anuradha *et al.* (2008 ▶, 2009 ▶); Chitra *et al.* (2009 ▶).
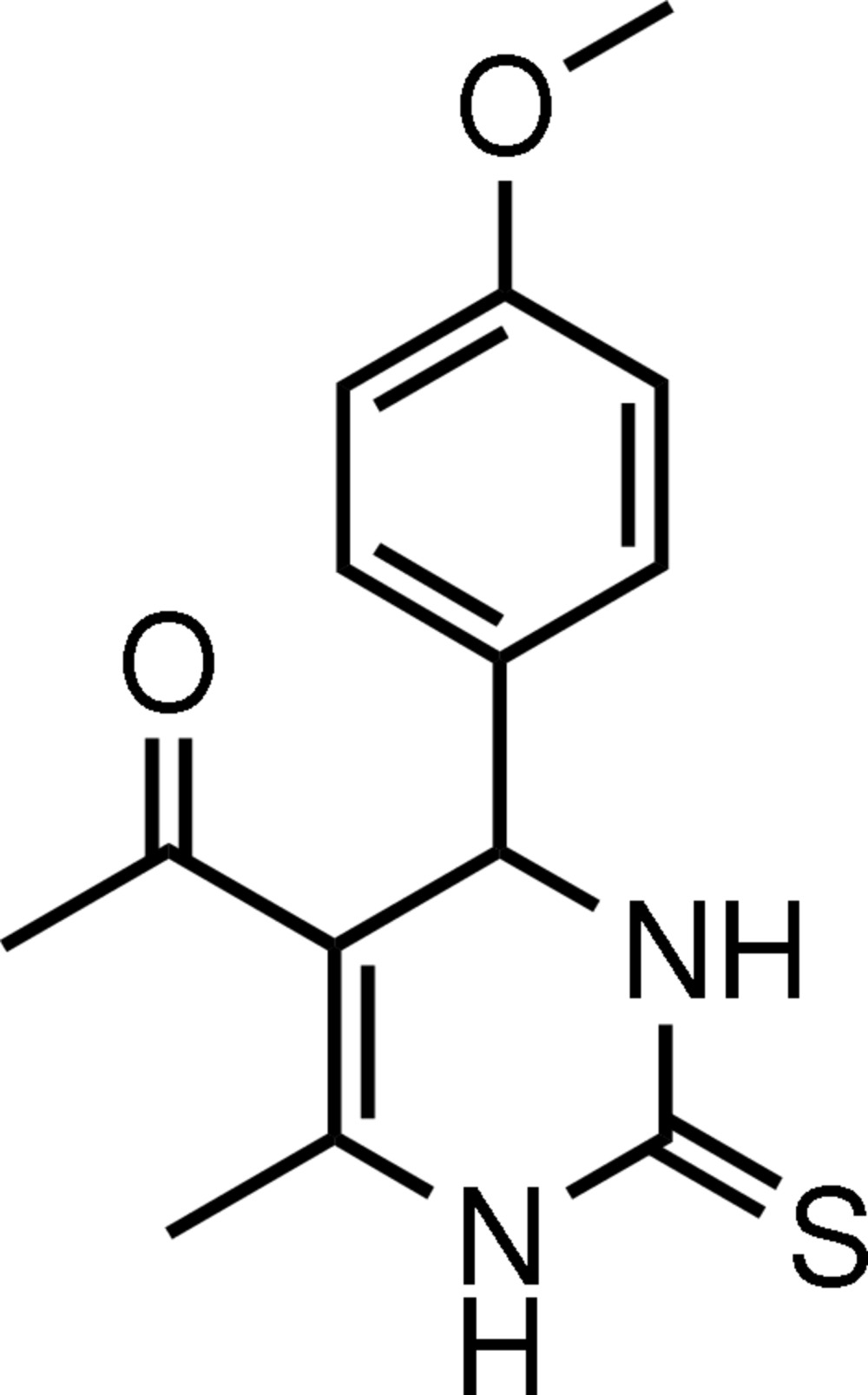


## Experimental

### 

#### Crystal data

C_14_H_16_N_2_O_2_S
*M* *_r_* = 276.36
Monoclinic, 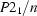

*a* = 12.0415 (2) Å
*b* = 6.2219 (1) Å
*c* = 18.0192 (3) Å
β = 100.901 (1)°
*V* = 1325.66 (4) Å^3^
*Z* = 4
Cu *K*α radiation
μ = 2.17 mm^−1^
*T* = 110 K
0.53 × 0.13 × 0.10 mm

#### Data collection

Oxford Diffraction Xcalibur Ruby Gemini diffractometer
Absorption correction: multi-scan (*CrysAlis Pro*; Oxford Diffraction, 2009 ▶) *T*_min_ = 0.418, *T*_max_ = 1.000
5263 measured reflections
2616 independent reflections
2447 reflections with *I* > 2σ(*I*)
*R*_int_ = 0.020

#### Refinement

*R*[*F* ^2^ > 2σ(*F* ^2^)] = 0.039
*wR*(*F* ^2^) = 0.112
*S* = 1.09
2616 reflections
183 parameters
H atoms treated by a mixture of independent and constrained refinement
Δρ_max_ = 0.44 e Å^−3^
Δρ_min_ = −0.28 e Å^−3^

### 

Data collection: *CrysAlis Pro* (Oxford Diffraction, 2009 ▶); cell refinement: *CrysAlis Pro*; data reduction: *CrysAlis Pro*; program(s) used to solve structure: *SHELXS97* (Sheldrick, 2008 ▶); program(s) used to refine structure: *SHELXL97* (Sheldrick, 2008 ▶); molecular graphics: *ORTEP-3* (Farrugia, 1997 ▶); software used to prepare material for publication: *PLATON* (Spek, 2009 ▶).

## Supplementary Material

Crystal structure: contains datablocks global, I. DOI: 10.1107/S160053680904639X/wn2363sup1.cif

Structure factors: contains datablocks I. DOI: 10.1107/S160053680904639X/wn2363Isup2.hkl

Additional supplementary materials:  crystallographic information; 3D view; checkCIF report

## Figures and Tables

**Table 1:** Hydrogen-bond geometry (Å, °)

*D*—H⋯*A*	*D*—H	H⋯*A*	*D*⋯*A*	*D*—H⋯*A*
N1—H1⋯S2^i^	0.87 (2)	2.61 (2)	3.464 (1)	169.3 (18)
N3—H3⋯O14^ii^	0.85 (2)	2.15 (2)	2.985 (2)	170 (2)
C16—H16*B*⋯O15^iii^	0.98	2.46	3.4377 (17)	176
C42—H42⋯O15^iv^	0.95	2.42	3.310 (2)	156
C61—H61*B*⋯S2^i^	0.98	2.80	3.7190 (14)	157

Symmetry codes: (i) 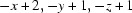; (ii) 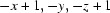; (iii) 
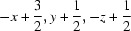
; (iv) 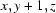.

**Fig. 1.**
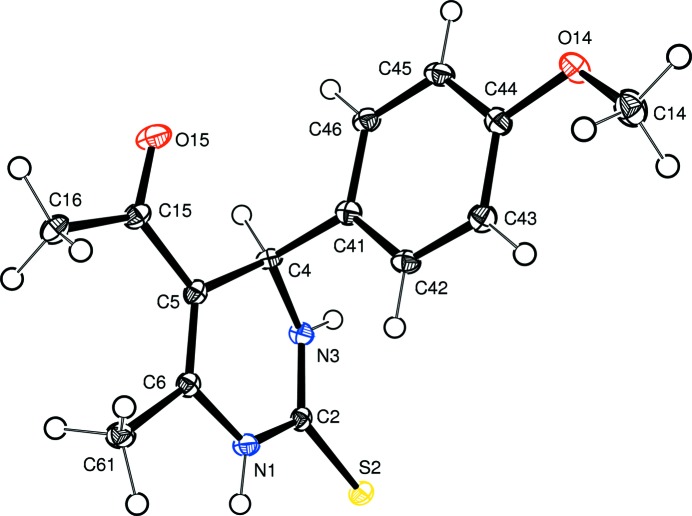
The molecular structure of the title compound, showing the atom-numbering scheme and displacement ellipsoids drawn at the 40% probability level. H atoms are shown as small spheres of arbitrary radius.

**Fig. 2.**
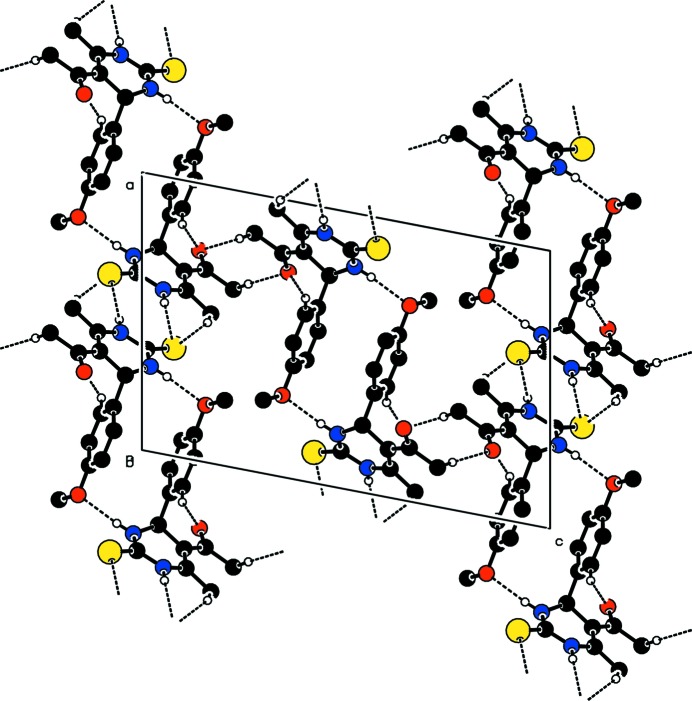
The packing of the title compound, viewed down the b axis. Dashed lines indicate hydrogen bonds. H atoms not involved in hydrogen bonding have been omitted.

## supplementary crystallographic information

### Comment

As part of our investigations of dihydropyrimidine derivatives to compare their biological activity, we have undertaken the X-ray crystal structure analysis of the title compound. The crystal structures of three very closely related compounds have recently been reported [Anuradha *et al.*, (2008, 2009); Chitra *et al.*, (2009]; these studies have also reported their chemical and biological applications.

In the title molecule, C_14_H_16_N_2_O_2_S, (Fig. 1), the heterocyclic ring adopts an envelope conformation with the plane through the five coplanar atoms (N1,C2,N3,C5,C6) making a dihedral angle of 88.99 (4)° with the benzene ring, which adopts an axial orientation. The thionyl, acetyl and methyl groups all have equatorial orientations. Intermolecular N1—H1···S2(2 - *x*, 1 - *y*, 1 - *z*), N3—H3···O14(1 - *x*, -*y*, 1 - *z*), C16—H16B···O15(3/2 - *x*, 1/2 + *y*, 1/2 - *z*), C42—H42···O15(*x*, 1 + *y*, *z*) and C61—H61B···S2(2 - *x*, 1 - *y*, 1 - *z*) hydrogen bonds are found in the crystal structure.

### Experimental

A solution of acetylacetone (1.001 g, 0.01 mol), 4-methoxybenzaldehyde (1.202 g, 0.01 mol) and thiourea (1.14 g, 0.015 mol) was heated under reflux in the presence of calcium fluoride (0.078 g, 0.001 mol) for 3 h (monitored by TLC). After completion of the reaction, the reaction mixture was cooled to room temperature and poured into crushed ice. The crude product containing also the catalyst was collected by filtration on a Buchner funnel. The mixture of the product and the catalyst was digested in methanol (40 ml). The undissolved catalyst was removed by filtration. The crude product was obtained by evaporation of the methanol and further purified by recrystallization from hot ethanol to afford the pure title compound. Yield 90% (1.2 g).

### Refinement

The two N-bound H atoms were located in a difference Fourier map and refined freely; N1—H1 = 0.87 (2) Å and N3—H3 = 0.85 (2) Å. The remaining H atoms were positioned geometrically and allowed to ride on their parent atoms, with C—H = 0.95 - 1.00 Å ; *U*_iso_(H) = k*U*_eq_(C), where k = 1.5 for methyl and 1.2 for all other H atoms.

### Crystal data

**Table:**

C_14_H_16_N_2_O_2_S	*F*(000) = 584
*M**_r_* = 276.36	*D*_x_ = 1.385 Mg m^−^^3^
Monoclinic, *P*2_1_/*n*	Melting point: 469.5 K
Hall symbol: -P 2yn	Cu *K*α radiation, λ = 1.54184 Å
*a* = 12.0415 (2) Å	Cell parameters from 4545 reflections
*b* = 6.2219 (1) Å	θ = 4.9–74.1°
*c* = 18.0192 (3) Å	µ = 2.17 mm^−^^1^
β = 100.901 (1)°	*T* = 110 K
*V* = 1325.66 (4) Å^3^	Needle, colorless
*Z* = 4	0.53 × 0.13 × 0.10 mm

### Data collection

**Table:**

Oxford Diffraction Xcalibur Ruby Gemini diffractometer	2616 independent reflections
Radiation source: fine-focus sealed tube	2447 reflections with *I* > 2σ(*I*)
graphite	*R*_int_ = 0.020
Detector resolution: 10.5081 pixels mm^-1^	θ_max_ = 74.1°, θ_min_ = 4.9°
ω scans	*h* = −12→15
Absorption correction: multi-scan (*CrysAlis PRO*; Oxford Diffraction, 2009)	*k* = −7→7
*T*_min_ = 0.418, *T*_max_ = 1.000	*l* = −21→22
5263 measured reflections	

### Refinement

**Table:**

Refinement on *F*^2^	Primary atom site location: structure-invariant direct methods
Least-squares matrix: full	Secondary atom site location: difference Fourier map
*R*[*F*^2^ > 2σ(*F*^2^)] = 0.039	Hydrogen site location: inferred from neighbouring sites
*wR*(*F*^2^) = 0.112	H atoms treated by a mixture of independent and constrained refinement
*S* = 1.09	*w* = 1/[σ^2^(*F*_o_^2^) + (0.0764*P*)^2^ + 0.4468*P*] where *P* = (*F*_o_^2^ + 2*F*_c_^2^)/3
2616 reflections	(Δ/σ)_max_ = 0.001
183 parameters	Δρ_max_ = 0.44 e Å^−^^3^
0 restraints	Δρ_min_ = −0.28 e Å^−^^3^

### Special details

**Table:**

Geometry. Bond distances, angles *etc*. have been calculated using the rounded fractional coordinates. All su's are estimated from the variances of the (full) variance-covariance matrix. The cell e.s.d.'s are taken into account in the estimation of distances, angles and torsion angles
Refinement. Refinement of *F*^2^ against ALL reflections. The weighted *R*-factor *wR* and goodness of fit *S* are based on *F*^2^, conventional *R*-factors *R* are based on *F*, with *F* set to zero for negative *F*^2^. The threshold expression of *F*^2^ > 2σ(*F*^2^) is used only for calculating *R*-factors(gt) *etc*. and is not relevant to the choice of reflections for refinement. *R*-factors based on *F*^2^ are statistically about twice as large as those based on *F*, and *R*- factors based on ALL data will be even larger.

### Fractional atomic coordinates and isotropic or equivalent isotropic displacement parameters (Å^2^)

**Table:**

	*x*	*y*	*z*	*U*_iso_*/*U*_eq_	
S2	0.88945 (3)	0.40406 (6)	0.57843 (2)	0.0167 (1)	
O14	0.29443 (9)	0.06284 (18)	0.34383 (6)	0.0192 (3)	
O15	0.74168 (10)	−0.43229 (18)	0.35876 (6)	0.0240 (3)	
N1	0.90803 (10)	0.2225 (2)	0.44765 (7)	0.0155 (3)	
N3	0.80719 (10)	0.0321 (2)	0.52096 (7)	0.0151 (3)	
C2	0.86586 (11)	0.2080 (2)	0.51219 (8)	0.0139 (4)	
C4	0.75819 (12)	−0.1161 (2)	0.45957 (8)	0.0140 (4)	
C5	0.82669 (12)	−0.1056 (2)	0.39675 (8)	0.0140 (4)	
C6	0.89493 (12)	0.0647 (2)	0.39170 (8)	0.0139 (4)	
C14	0.26371 (13)	0.2480 (3)	0.29726 (9)	0.0237 (4)	
C15	0.80594 (12)	−0.2907 (2)	0.34489 (8)	0.0158 (4)	
C16	0.85846 (12)	−0.3124 (2)	0.27577 (8)	0.0181 (4)	
C41	0.63430 (12)	−0.0628 (2)	0.43028 (8)	0.0148 (4)	
C42	0.60296 (12)	0.1390 (3)	0.40073 (8)	0.0162 (4)	
C43	0.49062 (12)	0.1883 (2)	0.37094 (8)	0.0169 (4)	
C44	0.40836 (12)	0.0309 (3)	0.37140 (8)	0.0159 (4)	
C45	0.43784 (12)	−0.1707 (3)	0.40213 (8)	0.0185 (4)	
C46	0.55023 (12)	−0.2172 (2)	0.43155 (8)	0.0172 (4)	
C61	0.96218 (13)	0.1116 (2)	0.33106 (8)	0.0178 (4)	
H1	0.9539 (17)	0.328 (3)	0.4448 (11)	0.022 (5)*	
H3	0.7815 (18)	0.021 (4)	0.5615 (12)	0.028 (5)*	
H4	0.76299	−0.26565	0.48025	0.0167*	
H14A	0.30923	0.25272	0.25759	0.0356*	
H14B	0.18337	0.23987	0.27404	0.0356*	
H14C	0.27750	0.37814	0.32827	0.0356*	
H16A	0.83989	−0.45371	0.25270	0.0271*	
H16B	0.82891	−0.19969	0.23937	0.0271*	
H16C	0.94075	−0.29797	0.29024	0.0271*	
H42	0.65940	0.24570	0.40083	0.0194*	
H43	0.47036	0.32683	0.35065	0.0202*	
H45	0.38117	−0.27642	0.40300	0.0222*	
H46	0.57023	−0.35485	0.45271	0.0206*	
H61A	0.91214	0.10624	0.28142	0.0267*	
H61B	0.99595	0.25503	0.33916	0.0267*	
H61C	1.02218	0.00414	0.33314	0.0267*	

### Atomic displacement parameters (Å^2^)

**Table:**

	*U*^11^	*U*^22^	*U*^33^	*U*^12^	*U*^13^	*U*^23^
S2	0.0187 (2)	0.0159 (2)	0.0155 (2)	−0.0015 (1)	0.0030 (1)	−0.0042 (1)
O14	0.0145 (5)	0.0235 (6)	0.0196 (5)	−0.0007 (4)	0.0030 (4)	0.0031 (4)
O15	0.0333 (7)	0.0176 (6)	0.0225 (5)	−0.0084 (4)	0.0087 (5)	−0.0039 (4)
N1	0.0175 (6)	0.0134 (6)	0.0161 (6)	−0.0030 (5)	0.0042 (5)	−0.0007 (5)
N3	0.0162 (6)	0.0175 (6)	0.0118 (5)	−0.0020 (5)	0.0034 (5)	−0.0011 (5)
C2	0.0127 (6)	0.0139 (7)	0.0140 (6)	0.0025 (5)	−0.0003 (5)	−0.0006 (5)
C4	0.0164 (7)	0.0112 (6)	0.0139 (6)	−0.0026 (5)	0.0020 (5)	−0.0002 (5)
C5	0.0152 (7)	0.0135 (7)	0.0128 (6)	0.0016 (5)	0.0014 (5)	−0.0003 (5)
C6	0.0136 (7)	0.0140 (7)	0.0135 (6)	0.0014 (5)	0.0012 (5)	0.0007 (5)
C14	0.0191 (7)	0.0262 (8)	0.0253 (8)	0.0031 (6)	0.0028 (6)	0.0052 (7)
C15	0.0184 (7)	0.0129 (7)	0.0149 (6)	0.0013 (5)	−0.0002 (5)	0.0003 (5)
C16	0.0222 (7)	0.0162 (7)	0.0154 (6)	−0.0001 (5)	0.0025 (5)	−0.0029 (5)
C41	0.0168 (7)	0.0167 (7)	0.0110 (6)	−0.0015 (5)	0.0030 (5)	−0.0012 (5)
C42	0.0173 (7)	0.0166 (7)	0.0151 (6)	−0.0042 (5)	0.0042 (5)	0.0004 (6)
C43	0.0193 (7)	0.0161 (7)	0.0159 (6)	0.0000 (5)	0.0048 (5)	0.0016 (5)
C44	0.0147 (7)	0.0205 (7)	0.0128 (6)	−0.0008 (5)	0.0032 (5)	−0.0014 (5)
C45	0.0176 (7)	0.0186 (8)	0.0200 (7)	−0.0056 (6)	0.0051 (5)	−0.0008 (6)
C46	0.0198 (7)	0.0143 (7)	0.0178 (7)	−0.0021 (5)	0.0046 (5)	0.0001 (5)
C61	0.0204 (7)	0.0170 (7)	0.0172 (7)	−0.0037 (5)	0.0067 (6)	−0.0014 (5)

### Geometric parameters (Å, °)

**Table:**

S2—C2	1.6927 (14)	C42—C43	1.392 (2)
O14—C14	1.432 (2)	C43—C44	1.394 (2)
O14—C44	1.3822 (18)	C44—C45	1.390 (3)
O15—C15	1.2290 (18)	C45—C46	1.387 (2)
N1—C2	1.3571 (19)	C4—H4	1.0000
N1—C6	1.3947 (18)	C14—H14A	0.9800
N3—C2	1.3282 (18)	C14—H14B	0.9800
N3—C4	1.4749 (18)	C14—H14C	0.9800
N1—H1	0.87 (2)	C16—H16A	0.9800
N3—H3	0.85 (2)	C16—H16B	0.9800
C4—C41	1.522 (2)	C16—H16C	0.9800
C4—C5	1.523 (2)	C42—H42	0.9500
C5—C6	1.3544 (19)	C43—H43	0.9500
C5—C15	1.4744 (19)	C45—H45	0.9500
C6—C61	1.507 (2)	C46—H46	0.9500
C15—C16	1.506 (2)	C61—H61A	0.9800
C41—C46	1.3989 (19)	C61—H61B	0.9800
C41—C42	1.388 (2)	C61—H61C	0.9800
			
S2···N1^i^	3.4641 (13)	C43···H14C	2.8000
S2···C16^ii^	3.6641 (15)	C43···H14A	2.7200
S2···H4^iii^	2.9400	C44···H3^vi^	2.80 (2)
S2···H1^i^	2.61 (2)	C45···H3^vi^	2.99 (2)
S2···H14C^iv^	3.1600	C61···H16B	2.8400
S2···H16C^ii^	2.9000	C61···H16C	2.6500
S2···H61B^i^	2.8000	H1···H61B	2.1100
S2···H61C^ii^	3.0800	H1···S2^i^	2.61 (2)
O14···C14^v^	3.181 (2)	H3···O14^vi^	2.15 (2)
O14···N3^vi^	2.9849 (16)	H3···C44^vi^	2.80 (2)
O15···N1^vii^	3.1588 (17)	H3···C45^vi^	2.99 (2)
O15···C42^vii^	3.310 (2)	H4···S2^vii^	2.9400
O15···C46	3.1575 (18)	H4···O15	2.3900
O15···C41	3.0411 (18)	H4···H46	2.3500
O14···H3^vi^	2.15 (2)	H14A···C43	2.7200
O14···H14A^v^	2.7900	H14A···H43	2.3600
O15···H4	2.3900	H14A···O14^ix^	2.7900
O15···H42^vii^	2.4200	H14C···C43	2.8000
O15···H16B^viii^	2.4600	H14C···H43	2.3000
O15···H61A^viii^	2.8500	H14C···S2^iv^	3.1600
N1···O15^iii^	3.1588 (17)	H16A···C15^viii^	3.0700
N1···S2^i^	3.4641 (13)	H16A···C42^viii^	3.0300
N3···O14^vi^	2.9849 (16)	H16A···H16B^viii^	2.5700
N3···H42	2.8600	H16B···C61	2.8400
C2···C42	3.440 (2)	H16B···H61A	2.2200
C2···C6^ii^	3.5055 (19)	H16B···O15^x^	2.4600
C6···C42	3.580 (2)	H16B···C42^viii^	2.9700
C6···C2^ii^	3.5055 (19)	H16B···H16A^x^	2.5700
C14···C14^v^	3.532 (3)	H16B···H42^viii^	2.5800
C14···C14^ix^	3.532 (3)	H16C···C6	3.0200
C14···O14^ix^	3.181 (2)	H16C···C61	2.6500
C16···C43^viii^	3.480 (2)	H16C···H61A	2.5400
C16···S2^ii^	3.6641 (15)	H16C···H61C	2.1900
C16···C61	3.0069 (18)	H16C···S2^ii^	2.9000
C16···C42^viii^	3.314 (2)	H42···O15^iii^	2.4200
C41···O15	3.0411 (18)	H42···N3	2.8600
C42···C6	3.580 (2)	H42···C2	2.8900
C42···O15^iii^	3.310 (2)	H42···C5	2.9800
C42···C16^x^	3.314 (2)	H42···C6	3.0800
C42···C2	3.440 (2)	H42···H16B^x^	2.5800
C43···C16^x^	3.480 (2)	H43···C14	2.5400
C46···O15	3.1575 (18)	H43···H14A	2.3600
C61···C16	3.0069 (18)	H43···H14C	2.3000
C2···H42	2.8900	H46···H4	2.3500
C5···H42	2.9800	H61A···C15	3.1000
C6···H16C	3.0200	H61A···C16	2.6800
C6···H42	3.0800	H61A···H16B	2.2200
C14···H43	2.5400	H61A···H16C	2.5400
C15···H16A^x^	3.0700	H61A···O15^x^	2.8500
C15···H61A	3.1000	H61B···H1	2.1100
C16···H61C	2.8400	H61B···S2^i^	2.8000
C16···H61A	2.6800	H61C···C16	2.8400
C42···H16B^x^	2.9700	H61C···H16C	2.1900
C42···H16A^x^	3.0300	H61C···S2^ii^	3.0800
			
C14—O14—C44	117.07 (12)	C41—C46—C45	120.48 (13)
C2—N1—C6	124.27 (12)	N3—C4—H4	108.00
C2—N3—C4	124.91 (12)	C5—C4—H4	108.00
C2—N1—H1	116.6 (13)	C41—C4—H4	108.00
C6—N1—H1	118.5 (13)	O14—C14—H14A	109.00
C2—N3—H3	117.3 (16)	O14—C14—H14B	109.00
C4—N3—H3	115.8 (16)	O14—C14—H14C	109.00
S2—C2—N3	122.14 (11)	H14A—C14—H14B	109.00
N1—C2—N3	116.77 (12)	H14A—C14—H14C	109.00
S2—C2—N1	121.08 (10)	H14B—C14—H14C	109.00
N3—C4—C5	109.55 (11)	C15—C16—H16A	109.00
N3—C4—C41	110.53 (11)	C15—C16—H16B	109.00
C5—C4—C41	111.47 (12)	C15—C16—H16C	109.00
C4—C5—C15	112.76 (11)	H16A—C16—H16B	109.00
C6—C5—C15	126.92 (13)	H16A—C16—H16C	109.00
C4—C5—C6	120.28 (12)	H16B—C16—H16C	109.00
N1—C6—C5	119.29 (13)	C41—C42—H42	119.00
N1—C6—C61	112.65 (11)	C43—C42—H42	119.00
C5—C6—C61	128.06 (12)	C42—C43—H43	121.00
C5—C15—C16	123.16 (12)	C44—C43—H43	121.00
O15—C15—C5	117.96 (13)	C44—C45—H45	120.00
O15—C15—C16	118.88 (12)	C46—C45—H45	120.00
C4—C41—C46	120.64 (11)	C41—C46—H46	120.00
C42—C41—C46	118.84 (13)	C45—C46—H46	120.00
C4—C41—C42	120.51 (12)	C6—C61—H61A	109.00
C41—C42—C43	121.41 (14)	C6—C61—H61B	109.00
C42—C43—C44	118.86 (14)	C6—C61—H61C	109.00
O14—C44—C43	123.70 (15)	H61A—C61—H61B	109.00
C43—C44—C45	120.54 (14)	H61A—C61—H61C	109.00
O14—C44—C45	115.74 (14)	H61B—C61—H61C	109.00
C44—C45—C46	119.84 (14)		
			
C14—O14—C44—C43	14.8 (2)	C4—C5—C6—N1	4.6 (2)
C14—O14—C44—C45	−166.54 (13)	C4—C5—C6—C61	−175.22 (13)
C6—N1—C2—S2	178.02 (11)	C15—C5—C6—N1	−177.58 (13)
C6—N1—C2—N3	−1.0 (2)	C15—C5—C6—C61	2.6 (2)
C2—N1—C6—C5	7.1 (2)	C4—C5—C15—O15	−2.64 (19)
C2—N1—C6—C61	−173.10 (13)	C4—C5—C15—C16	176.60 (12)
C4—N3—C2—S2	163.27 (11)	C6—C5—C15—O15	179.36 (14)
C4—N3—C2—N1	−17.7 (2)	C6—C5—C15—C16	−1.4 (2)
C2—N3—C4—C5	26.71 (18)	C4—C41—C42—C43	177.44 (13)
C2—N3—C4—C41	−96.51 (16)	C46—C41—C42—C43	−1.6 (2)
N3—C4—C5—C6	−19.06 (18)	C4—C41—C46—C45	−177.50 (13)
N3—C4—C5—C15	162.80 (11)	C42—C41—C46—C45	1.5 (2)
C41—C4—C5—C6	103.60 (15)	C41—C42—C43—C44	0.3 (2)
C41—C4—C5—C15	−74.54 (14)	C42—C43—C44—O14	179.52 (13)
N3—C4—C41—C42	59.07 (17)	C42—C43—C44—C45	1.0 (2)
N3—C4—C41—C46	−121.95 (14)	O14—C44—C45—C46	−179.68 (13)
C5—C4—C41—C42	−63.03 (16)	C43—C44—C45—C46	−1.0 (2)
C5—C4—C41—C46	115.95 (14)	C44—C45—C46—C41	−0.2 (2)

Symmetry codes: (i) −*x*+2, −*y*+1, −*z*+1; (ii) −*x*+2, −*y*, −*z*+1; (iii) *x*, *y*+1, *z*; (iv) −*x*+1, −*y*+1, −*z*+1; (v) −*x*+1/2, *y*−1/2, −*z*+1/2; (vi) −*x*+1, −*y*, −*z*+1; (vii) *x*, *y*−1, *z*; (viii) −*x*+3/2, *y*−1/2, −*z*+1/2; (ix) −*x*+1/2, *y*+1/2, −*z*+1/2; (x) −*x*+3/2, *y*+1/2, −*z*+1/2.

### Hydrogen-bond geometry (Å, °)

**Table:**

*D*—H···*A*	*D*—H	H···*A*	*D*···*A*	*D*—H···*A*
N1—H1···S2^i^	0.87 (2)	2.61 (2)	3.464 (1)	169.3 (18)
N3—H3···O14^vi^	0.85 (2)	2.15 (2)	2.985 (2)	170 (2)
C16—H16B···O15^x^	0.98	2.46	3.4377 (17)	176
C42—H42···O15^iii^	0.95	2.42	3.310 (2)	156
C61—H61B···S2^i^	0.98	2.80	3.7190 (14)	157

Symmetry codes: (i) −*x*+2, −*y*+1, −*z*+1; (vi) −*x*+1, −*y*, −*z*+1; (x) −*x*+3/2, *y*+1/2, −*z*+1/2; (iii) *x*, *y*+1, *z*.
